# Decomposing health inequality with population-based surveys: a case study in Rwanda

**DOI:** 10.1186/s12939-018-0769-1

**Published:** 2018-05-10

**Authors:** Kai Liu, Chunling Lu

**Affiliations:** 1 Department of Social Security, School of Labor and Human Resources, Renmin University of China, Haidian District, Beijing China; 2 Division of Global Health Equity, Brigham & Women’s Hospital, Boston, MA USA; 3 Department of Global Health and Social Medicine, Harvard Medical School, Boston, MA USA; 4 Department of Science and Technology-National Research Foundation (DST-NRF) Center of Excellence in Human Development, University of Witwatersrand, Johannesburg, South Africa

**Keywords:** Inequality, Medical care utilization, Household catastrophic health spending, Blinder-Oaxaca decomposition, Rwanda

## Abstract

**Background:**

Ensuring equal access to care and providing financial risk protection are at the center of the global health agenda. While Rwanda has made impressive progress in improving health outcomes, inequalities in medical care utilization and household catastrophic health spending (HCHS) between the impoverished and non-impoverished populations persist. Decomposing inequalities will help us understand the factors contributing to inequalities and design effective policy instruments in reducing inequalities. This study aims to decompose the inequalities in medical care utilization among those reporting illnesses and HCHS between the poverty and non-poverty groups in Rwanda.

**Methods:**

Using the 2005 and 2010 nationally representative Integrated Living Conditions Surveys, our analysis focuses on measuring contributions to inequalities from poverty status and other sources. We conducted multivariate logistic regression analysis to obtain poverty’s contribution to inequalities by controlling for all observed covariates. We used multivariate nonlinear decomposition method with logistic regression models to partition the relative and absolute contributions from other sources to inequalities due to compositional or response effects.

**Results:**

Poverty status accounted for the majority of inequalities in medical care utilization (absolute contribution 0.093 in 2005 and 0.093 in 2010) and HCHS (absolute contribution 0.070 in 2005 and 0.032 in 2010). Health insurance status (absolute contribution 0.0076 in 2005 and 0.0246 in 2010) and travel time to health centers (absolute contribution 0.0025 in 2005 and 0.0014 in 2010) were significant contributors to inequality in medical care utilization. Health insurance status (absolute contribution 0.0021 in 2005 and 0.0011 in 2010), having under-five children (absolute contribution 0.0012 in 2005 and 0.0011 in 2010), and having disabled family members (absolute contribution 0.0002 in 2005 and 0.0001 in 2010) were significant contributors to inequality in HCHS. Between 2005 and 2010, the main sources of the inequalities remained unchanged.

**Conclusions:**

Expanding insurance coverage and reducing travel time to health facilities for those living in poverty could be used as policy instruments to mitigate inequalities in medical care utilization and HCHS between the poverty and non-poverty groups.

**Electronic supplementary material:**

The online version of this article (10.1186/s12939-018-0769-1) contains supplementary material, which is available to authorized users.

## Background

Achieving health equity is at the center of global health agenda. The Sustainable Development Goals (SDGs) prioritizes improving equity over the next 15 years [1, 2]. Previous studies have identified significant inequalities in medical care utilization and catastrophic health spending between income groups in low- and middle-income countries such as Armenia, Burkina Faso, Indonesia, Vietnam, Chile, Turkey, China, India, Ghana, Tanzania, and Rwanda, with the poor less likely to use medical care and more likely to incur catastrophic health spending than those outside of poverty [3–13]. However, other than poverty status itself, other sources of inequalities between the income groups, and their exact contributions to such inequalities, remain unknown. To design effective interventions against inequalities between income groups and monitor progress in reducing inequalities, it is important to identify sources of inequalities between income levels and quantify their contributions to inequality. Drawing upon such evidence, policy makers and other stakeholders will be able to construct related short-term and long-term policy measures to effectively reach their targets.

Rwanda is a low-income agricultural country in central east Africa, with a gross domestic product per capita of US$690 in 2015 [14]. The country had a population of 11.3 million in 2014, with 83% of its population living in rural areas and 39.1% living below the national poverty line [15]. Since 2000, Rwanda has made impressive progress in improving health outcomes. Its under-five child mortality rate fell drastically, from 196 per 1000 live births in 2000 to 50 per 1000 live births in 2015 [16], making Rwanda one of only a few sub-Saharan countries that met the MDG target on reducing child mortality [17]. Previous studies have observed a significant increase in medical care utilization when in need and a significant reduction in households with catastrophic health spending (HCHS) in Rwanda [18–20]. However, inequalities in medical care utilization and HCHS between those living under poverty (poverty groups) and those living above poverty (non-poverty groups) have persisted over time (Liu K, Cook B, Lu C. Health inequality and community-based health insurance: a case study of rural Rwanda with repeated cross-sectional data, forthcoming).

Like many other developing countries, ensuring access to health care with financial risk protection for the poorest is part of the government’s policy agenda in Rwanda. Rwanda established *Mutuelles* at the national level in 2005 to promote health equity. In 2010, about 67% of individuals in Rwanda enrolled in the program (Liu K, Cook B, Lu C. Health inequality and community-based health insurance: a case study of rural Rwanda with repeated cross-sectional data, forthcoming). In both 2005 and 2010, the percentage of individuals enrolled in *Mutuelles* in the non-poverty group was significantly higher than in the poverty group, and the *Mutuelles* program did not play significant role in reducing inequalities in medical care utilization and HCHS between the two income groups in 2005 and 2010 (Liu K, Cook B, Lu C. Health inequality and community-based health insurance: a case study of rural Rwanda with repeated cross-sectional data, forthcoming).

In this study, we used the nationally-representative and publicly accessible Integrated Living Conditions Surveys (EICV) in 2005 and 2010 and decomposed the inequalities in medical care utilization among those reporting illnesses and HCHS between the poverty and non-poverty groups. We extended previous work by identifying the main sources of inequalities in addition to poverty status and quantifying their contributions in both compositional effect and response effect. We also tracked changes over time in the magnitude of their contributions.

## Methods

### Conceptual framework of decomposition of inequality between poverty and non-poverty groups

Health inequality between populations with different income levels is usually defined as the differences of observed means or medians for selected health indicators between these groups [17, 21, 22]. For example, the World Health Organization (WHO) published inequality measures for the coverage of immunization among under-five children by comparing the average rate of immunization between the poorest and the richest wealth quintiles [22]. The observed inequality in health indicators between the two income groups may originate from sources other than income itself. Abundant evidence has demonstrated that income level is not the only determinant for medical care utilization and HCHS. Other determinants include health need, education, insurance status, availability of quality service, preference, culture context, or other socio-demographic factors (such as age, gender, and race/ethnicity) [1–4, 8, 23]. These factors could confound the observed mean differences between income groups.

**Fig. 1:**
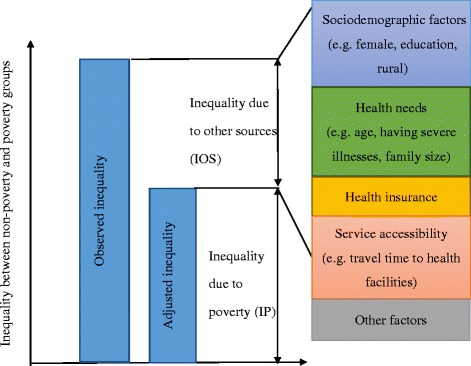
Decomposing the sources of inequality between poverty and non-poverty groups: an example of medical care utilization. The numerical size of the boxes of the five sub-segments of IOS is not proportional to their calculated size derived from the study findings

Figure 1 illustrates the contributors to the observed inequality in medical care utilization between the poverty and non-poverty groups: (1) poverty status, and (2) other sources, such as health insurance status, health care needs, etc. Inequality resulting from poverty status (IP) can be measured by adjusting observed variables using multivariate regression models. Inequality resulting from other sources (IOS) can be measured using the Blinder-Oaxaca decomposition method [24–26]. Observed inequality in medical care utilization between the non-poverty and poverty groups is the sum of IP and IOS. According to Blinder-Oaxaca decomposition method, the other sources contributing to inequality could be decomposed into two components: compositional effect and response effect. The compositional effect of a covariate comes from its between-group mean differences by poverty status, and the response effect results from different responses by poverty status to the covariate as well as the impact from unobserved factors (not included in the model). To use the covariate health insurance as an example, a positive coefficient of compositional effect for health insurance indicates the expected reduction in the poverty-non-poverty inequality gap if the poverty group was equal to the non-poverty group in the distribution of health insurance. A positive coefficient of response effect for health insurance indicates the expected decrease in the poverty-non-poverty inequality gap if the poverty group had the same return to health insurance as the non-poverty group.

### Data and sample

We used the repeated cross-sectional EICV household surveys in 2005 and 2010 [27]. The EICV measures medical care utilization, out-of-pocket health spending (OOPS), demographic and socioeconomic characteristics, health insurance status, and household income and expenditure. It is a nationally representative, repeated cross-sectional survey conducted in 2000, 2005, 2010, and 2014. The surveys in 2005 and 2010 provided consistent socioeconomic information, while those in 2000 and 2014 had problems with lack of key variables such as schooling and travel time to health center and changing definitions of poverty line, respectively. We therefore included only the analysis of 2005 and 2010. Details on its sampling and implementation process are presented in Additional file 1: Box S1 of the webappendix.

To estimate the sources of inequality in medical care utilization, we included only individuals who reported being ill two weeks before the survey. The final sample size was 6737 in 2005 and 11,944 in 2010, respectively. To estimate the sources of inequality in HCHS, we included all households in the two years, and the final sample size was 6639 in 2005 and 11,335 in 2010.

### Variables

#### Analysis on medical care utilization among those reporting illness

A dichotomous outcome variable was constructed to indicate medical care utilization among individuals who reported an illness in the previous two weeks of the survey. Medical services included outpatient services, inpatient services, and medical tests.

We constructed a dichotomous poverty indicator indicating whether a household lived above or below the national poverty line. In 2005 and 2010, 57 and 45% of households, respectively lived under the national poverty line which was defined the same way in both years [28]. In the EICV, there was no variable indicating whether a household lived under the national poverty line. Following previous studies [18, 19], we defined poverty households as those in the first, second and third wealth quintiles (60% of total households in the survey) in 2005 and the first and second wealth quintiles (40% of total households in the survey) in 2010, which were close to the percentage of households living under the national poverty line (60% vs. 57% in 2005, and 40% vs. 45% in 2010). We controlled for a number of demographic and socioeconomic variables including an individual’s age, gender, schooling of the household head, having severe illnesses, travel time to health center, health insurance status, household size, and geographic residence (Additional file 1: Box S2).

#### Analysis on HCHS

A dichotomous outcome variable was constructed to indicate whether or not a household had catastrophic health spending, defined as annual OOPS exceeding 40% of its annual capacity to pay [29, 30]. Additional file 1: Box S3 shows details on obtaining HCHS. To test the sensitivity of the results to the threshold of HCHS, we also constructed outcome variables using thresholds at the 10, 20, and 30% levels, respectively.

Other covariates included household head information (age, gender, and schooling), indicators on households having children under age five or disabled members, travel time to health center, health insurance status, household size, and geographic residence (Additional file 1: Box S2).

Summary statistics of used variables are presented in the Additional file 1: Tables S1 and S2.

### Statistical analysis

#### Calculating the observed and the adjusted mean differences between the non-poverty and poverty groups

Our decomposing analyses include two parts: (1) measuring IP, and (2) measuring IOS. Measuring IP allowed us to know the level of inequality between the non-poverty and poverty groups resulting from poverty status. To process the estimation, we first calculated the observed mean differences in medical care utilization and HCHS between the two income groups in 2005 and 2010. We set the poverty group as the comparison group and the non-poverty group as the reference group in the analysis. We then conducted multivariate logistic regression analysis to obtain the adjusted mean differences in the two health indicators between the two income groups (models are presented in Additional file 1: Box S4). By controlling for all observed covariates, the adjusted difference between the two income groups is due to poverty status. Percentage of inequality in medical care utilization and HCHS due to poverty was calculated as the adjusted mean difference divided by the observed mean difference between the two groups.

#### Estimating the contribution of covariates to inequality resulting from other sources (IOS)

The gap between the observed and adjusted mean differences between the two income groups was the inequality resulting from other sources (IOS). Measuring IOS enabled us to understand the contributions to inequality from other covariates, in addition to poverty status. We first conducted the t-test for each covariate to examine its mean difference by poverty status (Results of the t-test are shown in Additional file 1: Table S3).

We then used multivariate nonlinear decomposition method to partition the contribution of each covariate to the gap between the observed and adjusted inequalities in medical care utilization and HCHS. Multivariate decomposition has been widely used in quantifying the contributions of observed variables to group differences with multivariate regression models [24–26]. As the outcome variables are dichotomous, we adopted estimate method proposed by Powers, Yoshioka, and Yun [26]. Their method addressed issues such as sensitivity to the order of variables in entering decomposition and sensitivity to the choice of reference group for dichotomous covariates [26]. We processed the analysis with logistic regression models, adjusting for the sampling weight. The decomposition method allowed us to assess the magnitude of other sources contributing to inequality in terms of compositional effect and response effect. For example, in terms of health insurance status, we were able to determine the percentage of inequality in medical care utilization between the two income groups explained by (1) the mean difference of insurance coverage between the two income groups, as well as (2) the difference between the two income groups in medical care utilization responding to having insurance. For each covariate, we obtained its relative contribution to the IOS due to compositional or response effects using the statistical software program Stata. We then obtained its absolute contribution to the observed inequality by multiplying relative contribution to the IOS.

Stata version 14 was used for all analyses.

## Results

### Observed inequalities in medical care utilization and HCHS explained by poverty status in 2005 and 2010

#### Observed inequalities in medical care utilization

**Fig. 2:**
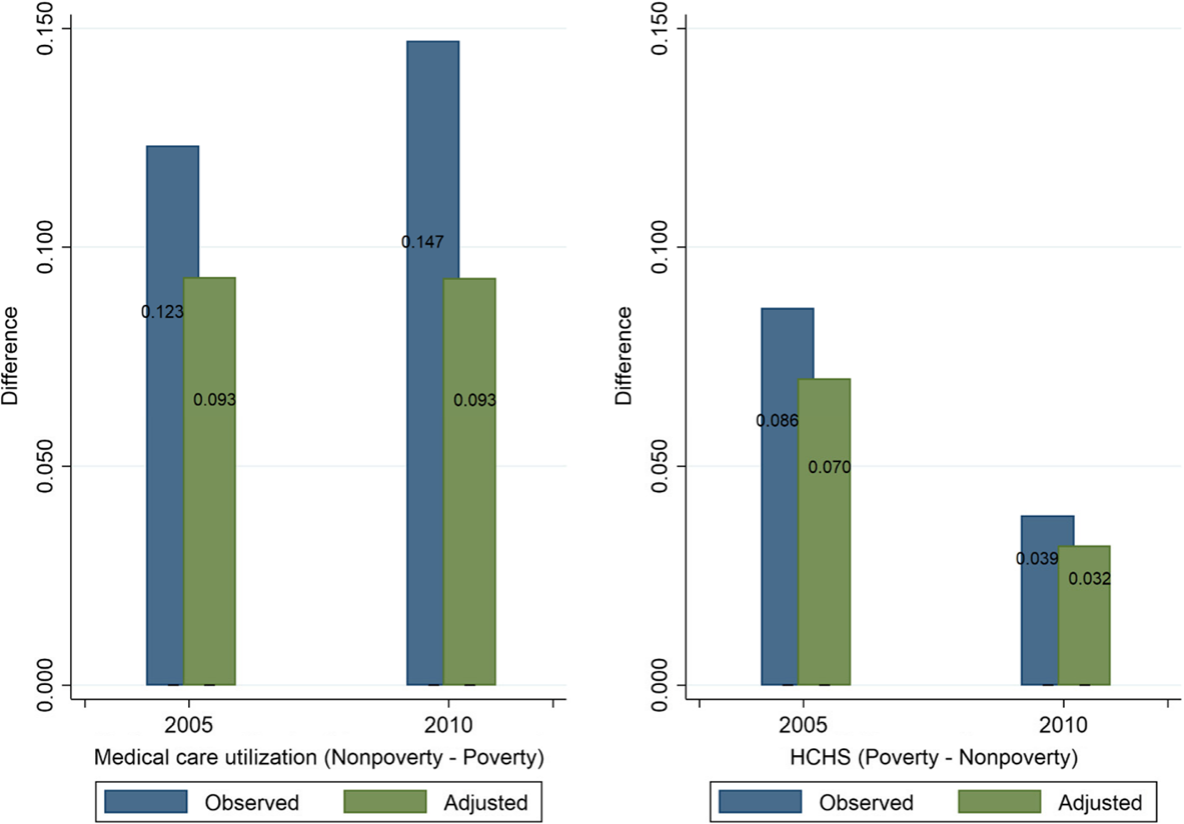
Absolute inequalities in medical care utilization and HCHS: Rwanda, 2005, 2010

The observed and adjusted differences in percentage of individuals using medical care between non-poverty and poverty groups were 0.123 and 0.093, respectively in 2005 and 0.147 and 0.093, respectively in 2010 (Fig. 2). Poverty status accounted for 76 and 63% of inequality in medical care utilization in 2005 and 2010 respectively. The results of the multivariate logistic model used to obtain the adjusted mean of medical care utilization are shown in Additional file 1: Table S4.

#### Observed inequalities in HCHS

The observed and adjusted differences in percentage of households with HCHS between poverty and non-poverty groups were 0.086 and 0.070, respectively in 2005 and 0.039 and 0.032, respectively in 2010 (Fig. 2). Poverty status accounted for 81 and 82% of inequality in HCHS in 2005 and 2010 respectively. Additional file 1: Figure S1 shows that results using the HCHS with the threshold of 40% were not sensitive to alternative thresholds (10, 20, and 30%) of the HCHS. The results of multivariate logistic model used to obtain the adjusted mean of HCHS are shown in Additional file 1: Table S5.

### Observed inequalities in medical care utilization explained by other variables in 2005 and 2010

#### Relative contribution of the compositional effects of covariates to IOS in medical care utilization

Table 1 presents the percentage of relative contribution of other sources to the gap between observed and adjusted inequalities (IOS) in medical care utilization using the Blinder-Oaxaca decomposition technique (Results of absolute contribution are shown in Additional file 1: Table S6). The compositional effects of all covariates accounted for 23.44 and 39.27% of the IOS in 2005 and 2010, respectively. Among all covariates, health insurance and travel time to health centers were the two major sources that had significant and positive contributions to the IOS. If the rate of insurance coverage in the poverty group was raised to the same level as that of the non-poverty group, the IOS would be reduced by 25.17 and 45.50% in 2005 and 2010, respectively. If average travel time to health centers in the poverty group was reduced to the same level as that of the non-poverty group, the IOS would be decreased by 8.36% in 2005 and 2.56% in 2010. Having severe illnesses and household size made inverse contributions to the IOS.

**Table 1:** Estimated relative contribution of covariates to inequalities in medical care utilization and HCHS by poverty status using BO decomposition method: Rwanda, 2005, 2010

	Inequality in medical care utilization		Inequality in HCHS	
	2005 (*N* = 6737)	2010 (*N* = 11,944)	2005 (*N* = 6639)	2010 (*N* = 11,335)
	Relative contribution (%)	Relative contribution (%)	Relative contribution (%)	Relative contribution (%)
Compositional effect				
Total	23.44***	39.27***	21.18***	17.33**
Female	0.02**	0.02**	−1.22**	1.15***
Head: no education	1.86	1.24	3.96*	1.75
Rural	−1.57	−2.22	5.18	−6.11*
Age group < 30	0.91***	0.35***	1.88	−1.46
Age group 30–50	0.10	0.27	1.97**	1.30
Age group > 50	−0.68**	−0.66***	0.05	0.35
Having severe illnesses	−7.13***	−2.78***		
Household having children			7.54***	15.59***
Household having disabled people			1.14***	1.81*
Household size	−3.69***	−4.94***	−8.87***	−6.98
Health insurance	25.17***	45.50***	8.45***	10.36***
Travel time to health center (> 0.5 h)	8.36***	2.56**	1.21	−0.44
Response effect				
Total	76.56***	60.71***	78.82***	82.67***
Female	11.91	6.58	−3.03	6.62
Head: no education	−7.03	−4.39	−3.37	5.61
Rural	−30.18	−20.24	27.72**	1.19
Age group < 30	−3.81	−5.22	−1.52	5.54
Age group 30–50	0.23	3.17	6.70	1.30
Age group > 50	0.65	−1.51	−3.37	−8.55
Having severe illnesses	−11.66	−6.85		
Household having children			−12.89	−9.01
Household having disabled people			3.41	−5.01
Household size	−39.03*	−32.98*	32.17	87.30***
Health insurance	−16.43*	−33.65***	13.20*	15.28
Travel time to health center (> 0.5 h)	14.95	−6.47	− 1.49	3.12
Constant	156.95***	162.30***	21.29	−20.71

*: statistically significant at the 0.10 level; **: statistically significant at the 0.05 level; ***: statistically significant at the 0.01 level

#### Relative contribution of the response effects of covariates to IOS in medical care utilization

The response effect of all covariates accounted for 76.56 and 60.71% of the IOS in 2005 and 2010, respectively. The constant term, which estimated the differential effects of variables not included in the model, was the largest contributor to the IOS. Among the covariates, health insurance made a significant inverse contribution to the IOS (at the 0.1 level in 2005 and 0.05 level in 2010), suggesting that the protective effects of health insurance were not as strong for the non-poverty group as it was for the poverty group. If the poverty group was protected by health insurance to the same degree as the non-poverty group, the IOS would be expected to increase by 16.43% in 2005 and 33.65% in 2010. This was also the case for household size: if the poverty group had the same returns to risk on household size as the non-poverty group, the IOS would increase by 39.03% in 2005 and 32.98% in 2010.

#### Absolute contribution of the compositional and response effects of covariates to IOS in medical care utilization

**Fig. 3:**
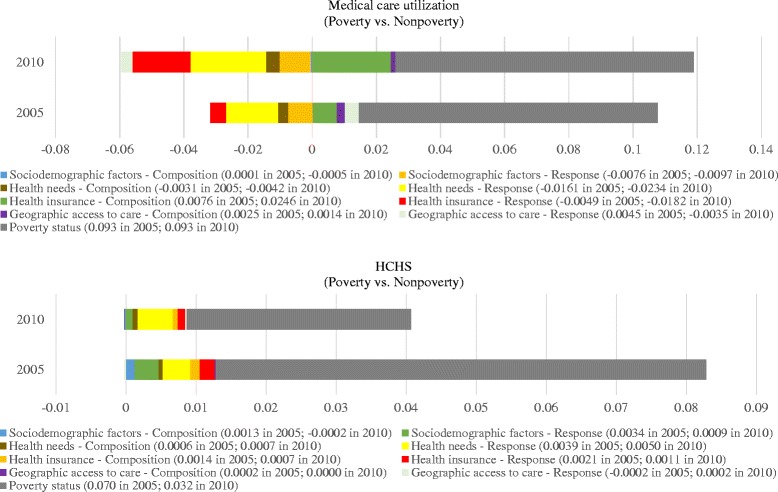
Decomposing absolute inequality in medical care utilization and HCHS by poverty status: Rwanda, 2005, 2010. “-Composition” represents compositional effect, and “-Response” represents response effect; the numbers in the bracket are absolute contributions to the inequalities, with the first number being in 2005 and the second in 2010; a negative value of the compositional effect for a covariate indicates the expected increase in the poverty-non-poverty inequality gap if the poverty group was equal to the non-poverty group in the distribution of the covariate; and a negative value of the response effect for a covariate indicates the expected increase in the poverty-non-poverty inequality gap if the poverty group had the same returns or risks to the covariate as did the non-poverty group

Figure 3 presents the absolute contribution of each source to the inequality in medical care utilization between the two income groups. For the convenience of graphing, we grouped covariates into four categories: 1) socio-demographic factors (gender, education, and geographic residence), 2) health needs (age, having severe illness, under-five child, disabled members, and household size), 3) health insurance, and 4) travel time to health center. The overall absolute contribution of a given category was equal to the sum of absolute contribution of all variables included in the category. While poverty status accounted for most of the inequalities in the two years, the other two major positive contributors were health insurance (compositional effect: 0.0076) and travel time to health centers (response effect: 0.0045) in 2005. In 2010, the order remained unchanged, with the compositional effect of travel time to health centers (0.0014) as the second main contributor.

### Observed inequalities in HCHS explained by other variables in 2005 and 2010

#### Relative contribution of the compositional effects of covariates to IOS in HCHS

Percentages of other sources contributing to the IOS in HCHS are presented in Table 1 (Results of absolute contribution are shown in Additional file 1: Table S7). The compositional effect of all covariates accounted for 21.18% of the IOS in 2005 and 17.33% in 2010. Among all covariates, health insurance and having under-five children and disabled people made significant positive contributions to the IOS in both years. Raising insurance enrollment rates in the poverty group to the same level as that of the non-poverty group would decrease the IOS in HCHS by 8.45% in 2005 and 10.36% in 2010. Reducing the percentage of household with under-five children or disabled people in the poverty group to the same level as that of the non-poverty group would lead to a decrease in the gap by 8.68% in 2005 and 17.40% in 2010.

#### Relative contribution of the response effects of covariates to IOS in HCHS

The response effect of covariates accounted for 78.82% of the IOS in 2005 and 82.67% in 2010. In 2005, the positive contributors include residence in rural areas and having health insurance: if living in rural areas or having health insurance affected the HCHS of the poverty group to the same extent as the non-poverty group, the IOS was expected to decrease by 27.7 and 13.20%, respectively. In 2010, household size had a significant and positive contribution to the IOS: if the poverty group was penalized by household size to the same extent as the non-poverty group, the IOS would be expected to decrease by 87.3%.

#### Absolute contribution of the compositional and response effects of covariates to IOS in HCHS

Between 2005 and 2010, the main positive contributors to inequality in HCHS remained unchanged (Fig. 3). Except for poverty status, health needs (response effect: 0.0039 in 2005 and 0.0050 in 2010), was the largest contributor among other covariates according to their absolute contribution. Socio-demographic factors (response effect: 0.0034) made greater contributions to the gap than did health insurance (response effect: 0.0021) in 2005, but less (response effect: 0.0009 versus 0.0011) in 2010.

## Discussion

Using the nationally representative EICV surveys in Rwanda in 2005 and 2010, this study has two salient findings. First, while poverty status was the largest contributor, other sources also made significant positive contributions to inequalities in medical care utilization (e.g., health insurance, travel time taken to health centers) and HCHS (e.g., health insurance, health needs) between the poverty and non-poverty groups. Second, the main sources of inequality in medical care utilization and HCHS remained unchanged between 2005 and 2010.

These findings study are consistent with previous studies about determinants of medical care utilization and HCHS in Rwanda. Evidence has shown that members of the poverty group were less likely to use medical care and more likely to incur HCHS [6, 18–20]. Enrolling in the *Mutuelles*, a community-based health insurance for rural residents and those in the informal economy in Rwanda, promoted medical care utilization and reduced catastrophic health spending in both 2005 and 2010 [6, 18]. Travel time taken to health centers was found to be inversely associated with medical care utilization, and health care needs were positively associated with medical care utilization and HCHS [6, 18]. In addition, previous studies found that health inequalities are associated with *Mutuelles* enrollment and benefit package design [20]. Differing from these studies, our study adds evidence about the exact contributions of other risk factors and poverty status itself to inequalities in medical care utilization and HCHS between income groups.

The positive coefficients of compositional effect for health insurance indicate that the reducing gaps in *Mutuelles* enrollment may potentially mitigate the inequalities in either medical care utilization or HCHS between the two income groups. In addition, *the Mutuelles* may not have provided the same protective function of promoting medical care utilization in the poverty group as it does in the non-poverty group in 2005 and 2010, as shown by the negative coefficient of response effect for health insurance. Both positive coefficient of compositional effect and negative coefficient of response effect for having health insurance suggest that, to mitigate inequalities between the two groups, policies for increasing *Mutuelles* enrollment among the poverty group and providing more protective effects to the poverty group (such as more service coverage) could be effective instruments. Since 2011, the Government of Rwanda has proposed providing a full subsidy for premiums and copayments *of the Mutuelles* for the poorest population in Rwanda (about 25%) [31], which could be expected to enlarge the coverage of *the Mutuelles* among the poorest and provide further financial risk protection to improve their access to care.

Between 2005 and 2010, accessibility of health services was substantially improved, with more health centers established during this time period (from 353 in 2005 to 436 in 2010). Numbers of physicians and nurses also increased, from 5,298 in 2005 to 8,806 in 2010 [18]. This improvement in service accessibility may explain the reduction of proportion of inequality in medical care utilization resulting from time travelling to health centers between 2005 and 2010.

Our study is subject to some potential limitations. First, factors (such as preferences, medical service availability, or satisfaction of services) that might contribute to observed inequalities in medical care utilization and HCHS between the two income groups were not included in the analysis due to unavailable data. As shown in this study and previous studies, the unknown factors could account for a sizable proportion of observed income-related inequality in medical care utilization [32]. Second, data were self-reported and may be subject to measurement errors, such as recall bias [33, 34]. Third, our construction of poverty indicators could potentially lead to under-estimation of inequality in 2005 and 2010, where 3% of non-poverty households were misallocated to the poverty group in 2005 and 5% of poverty households were misallocated to the non-poverty group in 2010.

## Conclusions

Findings from this study add to the knowledge of inequalities in medical care utilization and HCHS between the poverty and non-poverty groups in Rwanda. For the first time, the sources of inequalities were identified and their contributions were quantified in 2005 and 2010. Decomposing inequalities provided evidence for policy makers in designing interventions for reducing inequalities. In the long-term, eliminating poverty is a key solution to health care inequality and requires sustained economic growth and strong commitment from governments. In the short-term, as health insurance and travel time to health centers accounted for a considerable share of inequality between the poverty and non-poverty groups in medical care utilization and HCHS, expanding health insurance coverage and improving geographic access to health facilities for those living in poverty could be used as policy instruments for mitigating inequalities.

Future studies should focus on (1) evaluating the impact of policy instruments, such as eliminating premium and user fees of *Mutuelles* for those living in poverty, on reducing the inequalities between the poverty and non-poverty groups; and (2) identifying data sources in Rwanda that would allow us to analyze the confounding factors that were not included in this study and elucidate how they contributed to the observed inequalities in medical care utilization and HCHS.

## Additional file

Additional file 1: **Box S1.** Sampling and implementation process of the Integrated Living Conditions Survey (EICV). **Box S2.** Measurement of covariates used in the models on analyzing medical care utilization and catastrophic health spending. **Box S3.** Measurement of household catastrophic health spending (HCHS). **Box S4.** Multivariate logistic regression models in the analysis of adjusted mean of medical care utilization and HCHS. **Table S1.** Summary statistics for variables used in regression models on medical care utilization. **Table S2.** Summary statistics for variables used in regression models on HCHS. **Table S3.** T-tests about the mean differences of covariates by poverty status. **Table S4.** Odds ratios for covariates from the logistic model: Medical care utilization. **Table S5.** Odds ratios for covariates from the logistic model: HCHS. **Table S6.** Estimated absolute contribution of covariates to inequalities in medical care utilization by poverty status using BO decomposition method (EICV 2005, 2010). **Table S7.** Estimated contribution of covariates to inequalities in HCHS with different thresholds by poverty status using BO decomposition method (EICV 2005, 2010). **Figure S1.** Absolute inequalities in HCHS using different thresholds in 2005 and 2010. **Figure S2.** Decomposing absolute inequality in HCHS using different thresholds by poverty status in 2005 and 2010. (DOCX 89 kb)

## Abbreviations

EICV: Integrated Living Conditions Surveys
HCHS: Household catastrophic health spending
IOS: Inequality resulting from other sources
IP: Inequality resulting from poverty status
OOPS: Out-of-pocket health spending
SDGs: The Sustainable Development Goals
WHO: The World Health Organization
