# Supplementary material for: Decomposing health inequality with population-based surveys: a case study in Rwanda
Source: Int J Equity Health. 2018 May 10;17:57. doi: 10.1186/s12939-018-0769-1 (PMC5946429; doi:10.1186/s12939-018-0769-1)
Supplement: Supplementary file 1 — Box S1. Sampling and implementation process of the Integrated Living Conditions Survey (EICV). Box S2. Measurement of covariates used in the models on analyzing medical care utilization and catastrophic health spending. Box S3. Measurement of household catastrophic health spending (HCHS). Box S4. Multivariate logistic regression models in the analysis of adjusted mean of medical care utilization and HCHS. Table S1. Summary statistics for variables used in regression models on medical care utilization. Table S2. Summary statistics for variables used in regression models on HCHS. Table S3. T-tests about the mean differences of covariates by poverty status. Table S4. Odds ratios for covariates from the logistic model: Medical care utilization. Table S5. Odds ratios for covariates from the logistic model: HCHS. Table S6. Estimated absolute contribution of covariates to inequalities in medical care utilization by poverty status using BO decomposition method (EICV 2005, 2010). Table S7. Estimated contribution of covariates to inequalities in HCHS with different thresholds by poverty status using BO decomposition method (EICV 2005, 2010). Figure S1. Absolute inequalities in HCHS using different thresholds in 2005 and 2010. Figure S2. Decomposing absolute inequality in HCHS using different thresholds by poverty status in 2005 and 2010. (DOCX 89 kb) [file 12939_2018_769_MOESM1_ESM.docx]

**Additional Files**

**Box S1. Sampling and implementation process of the Integrated Living Conditions Survey (EICV)**

The EICV is a nationally representative, repeated cross-sectional survey conducted every five years starting in 2000. Each of the surveys lasted for 12 months, with the 2005 survey starting in January 2005 and ending in December 2005 and the 2010 survey commenced in November 2010 and finished in October 2011. There were 6,410, 6,900, and 14,308 households in the 2000, 2005, and 2010 EICV, respectively, with households being selected based on a stratified two-stage sample design. Sample villages were selected within each stratum (district) systematically with probability proportional to size at the first sampling stage and sample households were selected from each sample village at the second stage.^1^

**Box S2. Measurement of covariates used in the models on analyzing medical care utilization and catastrophic health spending**

*Covariates in the models of medical care utilization*

A categorical variable was constructed to identify individuals aged under 30, aged between 30 and 50, or aged over 50, with those aged under 30 serving as the reference group. Schooling of the household head was categorized into two groups: no schooling and having schooling. A dummy variable “having severe illnesses” was created, denoting whether or not an individual had to stay in bed during their latest illness over the course of the last two weeks. A dichotomous variable was constructed to indicate whether an individual participated in health insurance programs or was uninsured. In addition, we created a dummy variable, “travel time to health center,” to represent travel time of more than 0.5 hour to the nearest health center. Summary statistics for these variables are presented in supplementary Table A of the webappendix.

*Covariates in the models of catastrophic health spending*

Schooling of the household head, poverty status, travel time to health center, and household size were the same as those defined for individual-level utilization analysis. A categorical variable was constructed to identify the household head aged under 30, aged between 30 and 50, or aged over 50. Two dichotomous variables were created to indicate whether or not a household had under-five children and disabled people, respectively. A dichotomous variable was constructed to indicate whether a household participated in health insurance programs or was uninsured. Summary statistics for these variables is presented in supplementary Table B of the webappendix.

**Box S3. Measurement of household catastrophic health spending (HCHS)**

Using the WHO’s approach,^2,3^ we defined a household as having HCHS if its annual OOPS exceeds 40% of its annual capacity to pay. Capacity to pay was measured by household’s total expenditure, excluding spending on basic subsistence needs that were calculated as the average annual food expenditure of households whose food shares were in the 45th and 55th percentiles. The EICV includes questions about household spending on food, housing, education, durable goods, agriculture, and so on, making it possible to construct food spending (including self-made products and excluding alcohol, cigarettes, and restaurants) and total expenditure for each household. In addition, we calculated a household’s annual OOPS by including its spending on outpatient and inpatient services, drugs, medical tests, and transportation with a recall period of two weeks, as well as the spending on vaccination with a recall period of 12 months. We annualized items of OOPS with a two-week recall period by multiplying the value of each item by 26. To ensure the comparability of the estimates between years, we converted the value of OOPS and other expenditure-related items to the value of the Rwanda franc in 2010. For each household, the value of HCHS was “1” if the ratio of a household’s annual total OOPS to its capacity to pay was 0.4 or above, and “0” otherwise.

**Box S4. Multivariate logistic regression models in the analysis of adjusted mean of medical care utilization and HCHS**

To measure adjusted inequality in medical care utilization and HCHS between the two income groups, we processed the analysis with 2005 and 2010 data, respectively and used multivariate logistic regression models, as follows.

$Logit \left( {Utilization}/{HCHS} \right)=\beta_{0}+\beta_{1}{Poverty}\boldsymbol{+\beta}\boldsymbol{X}$ **(1)**

where Logit(Utilization/HCHS) represents the probability of using medical care or incurring HCHS, “Poverty” is poverty status of an individual or household. ***β*** is a vector of coefficients for **X** which is a vector of variables on individual or household characteristics.

**References**

1. Rwanda National Institute of Statistics. Integrated living conditions survey (2000, 2005, and 2010). <http://www.statistics.gov.rw/survey/integrated-household-living-conditions-survey-eicv> (accessed November 16, 2016).
2. Xu K, Evans DB, Kawabata K, et al. Household catastrophic health expenditure: a multicountry analysis. *Lancet* 2003; **362**: 111–17.
3. Xu K, Evans D, Carrin G, et al. Protecting households from catastrophic health spending. *Health Aff (Millwood)* 2007; **26**: 972–83.
4. Powers DA, Yoshioka H, Yun M-S. mvdcmp: multivariate decomposition for nonlinear response models. Stata J. 2011;11(4):556–76.

**Table S1. Summary statistics for variables used in regression models on medical care utilization**

|  | 2005 | | | 2010 | | |
| --- | --- | --- | --- | --- | --- | --- |
|  | N | Mean | SD | N | Mean | SD |
| Medical care utilization | 6,737 | 0.318 | 0.466 | 11,944 | 0.391 | 0.488 |
| Poverty | 6,737 | 0.569 | 0.495 | 11,944 | 0.394 | 0.489 |
| Age group <30 | 6,737 | 0.661 | 0.473 | 11,944 | 0.614 | 0.487 |
| Age group 30–50 | 6,737 | 0.202 | 0.401 | 11,944 | 0.205 | 0.403 |
| Age group >50 | 6,737 | 0.137 | 0.344 | 11,944 | 0.182 | 0.386 |
| Having severe illnesses | 6,725 | 0.720 | 0.449 | 11,937 | 0.498 | 0.500 |
| Household size | 6,737 | 5.701 | 2.386 | 11,944 | 5.371 | 2.198 |
| Female | 6,737 | 0.560 | 0.496 | 11,944 | 0.584 | 0.493 |
| Head: no education | 6,737 | 0.307 | 0.461 | 11,940 | 0.275 | 0.446 |
| Rural | 6,737 | 0.787 | 0.409 | 11,944 | 0.852 | 0.355 |
| Health insurance (individual) | 6,737 | 0.398 | 0.490 | 11,944 | 0.679 | 0.467 |
| Travel time to health center (>0.5 hour) | 6,737 | 0.720 | 0.449 | 11,944 | 0.696 | 0.460 |

**Table S2. Summary statistics for variables used in regression models on HCHS**

|  | 2005 | | | 2010 | | |
| --- | --- | --- | --- | --- | --- | --- |
|  | N | Mean | SD | N | Mean | SD |
| HCHS10% | 6,639 | 0.185 | 0.389 | 11,335 | 0.066 | 0.248 |
| HCHS20% | 6,639 | 0.130 | 0.336 | 11,335 | 0.043 | 0.202 |
| HCHS30% | 6,639 | 0.098 | 0.298 | 11,335 | 0.034 | 0.182 |
| HCHS40% | 6,639 | 0.080 | 0.272 | 11,335 | 0.029 | 0.169 |
| Poverty | 6,639 | 0.559 | 0.496 | 11,335 | 0.378 | 0.485 |
| Head: age group <30 | 6,639 | 0.223 | 0.416 | 11,335 | 0.213 | 0.410 |
| Head: age group 30–50 | 6,639 | 0.473 | 0.499 | 11,335 | 0.443 | 0.497 |
| Head: age group >50 | 6,639 | 0.304 | 0.460 | 11,335 | 0.344 | 0.475 |
| Household having children | 6,639 | 0.590 | 0.492 | 11,335 | 0.566 | 0.496 |
| Household having disabled people | 6,639 | 0.162 | 0.369 | 11,335 | 0.182 | 0.386 |
| Household size | 6,639 | 4.966 | 2.294 | 11,335 | 4.645 | 2.136 |
| Head: female | 6,639 | 0.287 | 0.452 | 11,335 | 0.291 | 0.454 |
| Head: no education | 6,639 | 0.316 | 0.465 | 11,333 | 0.286 | 0.452 |
| Rural | 6,639 | 0.783 | 0.412 | 11,335 | 0.861 | 0.346 |
| Health insurance (household) | 6,639 | 0.437 | 0.496 | 11,335 | 0.656 | 0.475 |
| Travel time to health center (>0.5 hour) | 6,639 | 0.706 | 0.456 | 11,335 | 0.707 | 0.455 |

**Table S3. T-tests about the mean differences of covariates by poverty status**

|  | 2005 | | | 2010 | | |
| --- | --- | --- | --- | --- | --- | --- |
|  | Non-poverty | Poverty | P value | Non-poverty | Poverty | P value |
| *Medical care utilization* | | | | | | |
| Age group <30 | 0.677 | 0.650 | 0.021 | 0.614 | 0.612 | 0.798 |
| Age group 30–50 | 0.179 | 0.219 | 0.000 | 0.192 | 0.223 | 0.000 |
| Age group >50 | 0.144 | 0.131 | 0.130 | 0.193 | 0.165 | 0.000 |
| Having severe illnesses | 0.696 | 0.737 | 0.000 | 0.492 | 0.505 | 0.170 |
| Household size | 5.587 | 5.788 | 0.001 | 5.134 | 5.737 | 0.000 |
| Female | 0.561 | 0.560 | 0.952 | 0.582 | 0.585 | 0.751 |
| Head: no education | 0.213 | 0.378 | 0.000 | 0.231 | 0.342 | 0.000 |
| Rural | 0.651 | 0.891 | 0.000 | 0.805 | 0.925 | 0.000 |
| Health insurance (individual) | 0.489 | 0.330 | 0.000 | 0.761 | 0.553 | 0.000 |
| Travel time to health center (>0.5 hour) | 0.159 | 0.243 | 0.000 | 0.131 | 0.200 | 0.000 |
| N | 6,737 | | | 11,944 | | |
| *HCHS* | | | | | | |
| Head: age group <30 | 0.295 | 0.167 | 0.000 | 0.251 | 0.151 | 0.000 |
| Head: age group 30–50 | 0.418 | 0.516 | 0.000 | 0.398 | 0.516 | 0.000 |
| Head: age group >50 | 0.287 | 0.317 | 0.008 | 0.350 | 0.333 | 0.061 |
| Household having children | 0.536 | 0.634 | 0.000 | 0.504 | 0.667 | 0.000 |
| Household having disabled people | 0.140 | 0.180 | 0.000 | 0.165 | 0.211 | 0.000 |
| Household size | 4.619 | 5.239 | 0.000 | 4.289 | 5.232 | 0.000 |
| Head: female | 0.265 | 0.303 | 0.001 | 0.285 | 0.302 | 0.044 |
| Head: no education | 0.217 | 0.394 | 0.000 | 0.247 | 0.351 | 0.000 |
| Rural | 0.638 | 0.897 | 0.000 | 0.817 | 0.933 | 0.000 |
| Health insurance (household) | 0.501 | 0.386 | 0.000 | 0.721 | 0.548 | 0.000 |
| Travel time to health center (>0.5 hour) | 0.152 | 0.217 | 0.000 | 0.135 | 0.196 | 0.000 |
| N | 6,639 | | | 11,335 | | |

T-tests results on medical care utilization show that, compared to those in non-poverty group, individuals in the poverty group had significant higher average rates in household size, household head with no education, living rural areas, having no health insurance, more travel time to health centers, and between age 30 and 50. There was no significant difference in gender between the two income groups. T-tests on HCHS show that, compared to non-poverty group, households in the poverty group had significant higher average values in age of head, female head, having children under five, disabled people, household size, living in rural areas, and travel time to health center; and had significant lower average values in having education and enrolling in health insurance.

**Table S4. Odds ratios for covariates from the logistic model: Medical care utilization**

|  | 2005 | 2010 |
| --- | --- | --- |
|  | OR (95% CI) | OR (95% CI) |
| Poverty | 0.64 (0.57,0.73)*** | 0.67 (0.61,0.74)*** |
| Female | 0.87 (0.78,0.98)* | 0.99 (0.91,1.09) |
| Head: no education | 0.86 (0.76,0.99)* | 0.85 (0.76,0.95)** |
| Rural | 0.89 (0.76,1.04) | 1.00 (0.88,1.13) |
| Age group <30 (Ref.) | 1.00 (Ref.) | 1.00 (Ref.) |
| Age group 30–50 | 0.80 (0.69,0.93)** | 0.79 (0.71,0.89)*** |
| Age group >50 | 0.66 (0.55,0.79)*** | 0.60 (0.53,0.68)*** |
| Having severe illnesses | 2.96 (2.56,3.42)*** | 2.62 (2.39,2.87)*** |
| Household size | 1.05 (1.02,1.08)*** | 1.03 (1.01,1.05)* |
| Health insurance (individual) | 2.41 (2.14,2.71)*** | 4.92 (4.40,5.52)*** |
| Travel time to health center (>0.5 hour) | 0.62 (0.55,0.71)*** | 0.79 (0.72,0.87)*** |

*: statistically significant at the 0.05 level; **: statistically significant at the 0.01 level; ***: statistically significant at the 0.001 level; OR: odds ratio; CI: confidence interval.

**Table 5S. Odds ratios for covariates from the logistic model: HCHS**

|  | 2005 | 2010 |
| --- | --- | --- |
|  | OR (95% CI) | OR (95% CI) |
| *HCHS_10%* |  |  |
| Poverty | 1.40 (1.21,1.63)*** | 1.44 (1.21,1.71)*** |
| Head: female | 0.94 (0.79,1.11) | 1.34 (1.11,1.63)** |
| Head: no education | 1.14 (0.98,1.34) | 1.01 (0.83,1.24) |
| Rural | 0.90 (0.75,1.08) | 0.66 (0.53,0.83)*** |
| Head: age group <30 | 1.00 (1.00,1.00) | 1.00 (1.00,1.00) |
| Head: age group 30–50 | 1.13 (0.93,1.36) | 1.02 (0.81,1.28) |
| Head: age group >50 | 0.97 (0.77,1.21) | 0.98 (0.75,1.29) |
| Household having children | 1.82 (1.52,2.17)*** | 1.89 (1.56,2.28)*** |
| Household having disabled people | 1.33 (1.12,1.59)** | 1.54 (1.26,1.89)*** |
| Household size | 0.97 (0.93,1.00) | 0.92 (0.87,0.97)*** |
| Health insurance (household) | 0.55 (0.48,0.64)*** | 0.57 (0.48,0.68)*** |
| Travel time to health center (>0.5 hour) | 1.15 (0.99,1.35) | 1.02 (0.85,1.23) |
| *HCHS_20%* |  |  |
| Poverty | 2.07 (1.72,2.49)*** | 2.17 (1.76,2.66)*** |
| Head: female | 0.80 (0.66,0.98)* | 1.39 (1.10,1.75)** |
| Head: no education | 1.20 (1.01,1.44)* | 1.03 (0.80,1.32) |
| Rural | 0.90 (0.72,1.13) | 0.69 (0.52,0.90)** |
| Head: age group <30 | 1.00 (1.00,1.00) | 1.00 (1.00,1.00) |
| Head: age group 30–50 | 1.12 (0.90,1.38) | 1.11 (0.84,1.47) |
| Head: age group >50 | 0.96 (0.74,1.25) | 1.02 (0.73,1.44) |
| Household having children | 1.83 (1.49,2.25)*** | 2.07 (1.64,2.61)*** |
| Household having disabled people | 1.41 (1.16,1.71)*** | 1.65 (1.28,2.11)*** |
| Household size | 0.91 (0.87,0.96)*** | 0.87 (0.82,0.93)*** |
| Health insurance (household) | 0.48 (0.41,0.57)*** | 0.52 (0.42,0.63)*** |
| Travel time to health center (>0.5 hour) | 1.03 (0.86,1.23) | 0.94 (0.75,1.18) |
| *HCHS_30%* |  |  |
| Poverty | 2.59 (2.08,3.23)*** | 2.71 (2.15,3.43)*** |
| Head: female | 0.78 (0.63,0.97)* | 1.53 (1.18,1.98)** |
| Head: no education | 1.32 (1.08,1.61)** | 1.04 (0.79,1.38) |
| Rural | 1.09 (0.83,1.44) | 0.64 (0.48,0.87)** |
| Head: age group <30 | 1.00 (1.00,1.00) | 1.00 (1.00,1.00) |
| Head: age group 30–50 | 1.29 (1.01,1.65)* | 1.03 (0.75,1.41) |
| Head: age group >50 | 1.09 (0.80,1.47) | 0.96 (0.65,1.43) |
| Household having children | 2.13 (1.68,2.69)*** | 2.21 (1.71,2.85)*** |
| Household having disabled people | 1.33 (1.07,1.67)* | 1.61 (1.22,2.13)*** |
| Household size | 0.88 (0.83,0.92)*** | 0.86 (0.80,0.93)*** |
| Health insurance (household) | 0.48 (0.39,0.58)*** | 0.55 (0.44,0.69)*** |
| Travel time to health center (>0.5 hour) | 1.05 (0.85,1.30) | 0.94 (0.73,1.20) |
| *HCHS_40%* |  |  |
| Poverty | 3.47 (2.68,4.50)*** | 3.42 (2.62,4.47)*** |
| Head: female | 0.79 (0.62,1.00) | 1.50 (1.13,1.99)** |
| Head: no education | 1.30 (1.05,1.62)* | 1.05 (0.77,1.42) |
| Rural | 1.03 (0.75,1.40) | 0.67 (0.48,0.93)* |
| Head: age group <30 | 1.00 (1.00,1.00) | 1.00 (1.00,1.00) |
| Head: age group 30–50 | 1.25 (0.95,1.63) | 1.14 (0.80,1.61) |
| Head: age group >50 | 1.10 (0.79,1.53) | 1.00 (0.65,1.55) |
| Household having children | 2.26 (1.75,2.92)*** | 2.14 (1.62,2.81)*** |
| Household having disabled people | 1.36 (1.07,1.74)* | 1.60 (1.18,2.16)** |
| Household size | 0.87 (0.82,0.92)*** | 0.85 (0.78,0.92)*** |
| Health insurance (household) | 0.49 (0.39,0.61)*** | 0.55 (0.42,0.70)*** |
| Travel time to health center (>0.5 hour) | 1.12 (0.89,1.41) | 0.94 (0.72,1.23) |

*: statistically significant at the 0.05 level; **: statistically significant at the 0.01 level; ***: statistically significant at the 0.001 level; OR: odds ratio; CI: confidence interval.

**Table S6. Estimated absolute contribution of covariates to inequalities in medical care utilization by poverty status using BO decomposition method (EICV 2005, 2010)**

|  | 2005 (N=6,737) | | 2010 (N=11,944) | |
| --- | --- | --- | --- | --- |
|  | Absolute contribution | P value | Absolute contribution | P value |
| Total | 0.0070 | 0.000 | 0.0212 | 0.000 |
| Socio-demographic factors | 0.0001 |  | -0.0005 |  |
| Female | 0.0000 | 0.016 | 0.0000 | 0.490 |
| Head: no education | 0.0006 | 0.383 | 0.0007 | 0.377 |
| Rural | -0.0005 | 0.702 | -0.0012 | 0.265 |
| Health needs | -0.0031 |  | -0.0042 |  |
| Age group <30 | 0.0003 | 0.000 | 0.0002 | 0.000 |
| Age group 30–50 | 0.0000 | 0.844 | 0.0001 | 0.322 |
| Age group >50 | -0.0002 | 0.017 | -0.0004 | 0.003 |
| Having severe illnesses | -0.0021 | 0.000 | -0.0015 | 0.000 |
| Household size | -0.0011 | 0.000 | -0.0027 | 0.008 |
| Health insurance (individual) | 0.0076 | 0.000 | 0.0246 | 0.000 |
| Travel time to health center (>0.5 hour) | 0.0025 | 0.000 | 0.0014 | 0.041 |
| Total | 0.0230 | 0.000 | 0.0328 | 0.000 |
| Socio-demographic factors | -0.0076 |  | -0.0097 |  |
| Female | 0.0036 | 0.245 | 0.0036 | 0.396 |
| Head: no education | -0.0021 | 0.171 | -0.0024 | 0.206 |
| Rural | -0.0091 | 0.139 | -0.0109 | 0.230 |
| Health needs | -0.0161 |  | -0.0234 |  |
| Age group <30 | -0.0011 | 0.672 | -0.0028 | 0.341 |
| Age group 30–50 | 0.0001 | 0.937 | 0.0017 | 0.135 |
| Age group >50 | 0.0002 | 0.821 | -0.0008 | 0.509 |
| Having severe illnesses | -0.0035 | 0.459 | -0.0037 | 0.278 |
| Household size | -0.0117 | 0.061 | -0.0178 | 0.074 |
| Health insurance (individual) | -0.0049 | 0.065 | -0.0182 | 0.002 |
| Travel time to health center (>0.5 hour) | 0.0045 | 0.276 | -0.0035 | 0.477 |
| Constant | 0.0471 | 0.000 | 0.0876 | 0.000 |

**Table S7. Estimated contribution of covariates to inequalities in HCHS with different thresholds by poverty status using BO decomposition method (EICV 2005, 2010)**

|  | 2005 (N=6,639) | | | 2010 (N=11,335) | | | | |
| --- | --- | --- | --- | --- | --- | --- | --- | --- |
|  | Relative contribution (%) | Absolute contribution | P value | Relative contribution (%) | Absolute contribution | | P value | |
| *HCHS_10%* | | | | | | | | |
| Compositional effect | | | | | | | | |
| Total | 35.11 | 0.0074 | 0.000 | 39.98 | 0.0032 | | 0.001 | |
| Socio-demographic factors | 3.11 | 0.0007 |  | -3.82 | -0.0003 | |  | |
| Head: female | -1.78 | -0.0004 | 0.074 | 1.53 | 0.0001 | | 0.005 | |
| Head: no education | 3.72 | 0.0008 | 0.282 | 1.62 | 0.0001 | | 0.619 | |
| Rural | 1.18 | 0.0002 | 0.846 | -6.97 | -0.0006 | | 0.228 | |
| Health needs | 14.32 | 0.0030 |  | 25.20 | 0.0020 | |  | |
| Head: age group <30 | 0.50 | 0.0001 | 0.840 | 0.81 | 0.0001 | | 0.799 | |
| Head: age group 30–50 | 3.20 | 0.0007 | 0.034 | 2.12 | 0.0002 | | 0.403 | |
| Head: age group >50 | -0.42 | -0.0001 | 0.144 | 0.14 | 0.0000 | | 0.685 | |
| Household having children | 9.91 | 0.0021 | 0.000 | 24.59 | 0.0020 | | 0.000 | |
| Household having disabled people | 1.83 | 0.0004 | 0.010 | 3.42 | 0.0003 | | 0.034 | |
| Household size | -0.69 | -0.0001 | 0.851 | -5.88 | -0.0005 | | 0.485 | |
| Health insurance (household) | 13.06 | 0.0027 | 0.000 | 19.69 | 0.0016 | | 0.000 | |
| Travel time to health center (>0.5 hour) | 4.61 | 0.0010 | 0.039 | -1.11 | -0.0001 | | 0.766 | |
| Response effect | | | | | | | | |
| Total | 64.90 | 0.0136 | 0.000 | 60.02 | 0.0048 | | 0.003 | |
| Socio-demographic factors | 6.01 | 0.0013 |  | 48.40 | 0.0039 | |  | |
| Head: female | -17.11 | -0.0036 | 0.035 | 6.78 | 0.0005 | | 0.472 | |
| Head: no education | -4.45 | -0.0009 | 0.529 | 5.62 | 0.0004 | | 0.519 | |
| Rural | 27.57 | 0.0058 | 0.242 | 36.00 | 0.0029 | | 0.261 | |
| Health needs | 30.27 | 0.0064 |  | 68.20 | 0.0055 | |  | |
| Head: age group <30 | 1.83 | 0.0004 | 0.772 | -1.65 | -0.0001 | | 0.808 | |
| Head: age group 30–50 | 6.54 | 0.0014 | 0.357 | 6.82 | 0.0005 | | 0.391 | |
| Head: age group >50 | -6.68 | -0.0014 | 0.306 | -3.71 | -0.0003 | | 0.664 | |
| Household having children | -36.54 | -0.0077 | 0.019 | -9.02 | -0.0007 | | 0.583 | |
| Household having disabled people | -1.09 | -0.0002 | 0.805 | -7.76 | -0.0006 | | 0.150 | |
| Household size | 66.22 | 0.0139 | 0.026 | 83.53 | 0.0067 | | 0.016 | |
| Health insurance (household) | 33.38 | 0.0070 | 0.005 | 22.62 | 0.0018 | | 0.250 | |
| Travel time to health center (>0.5 hour) | 19.23 | 0.0040 | 0.292 | -12.25 | -0.0010 | | 0.555 | |
| Constant | -24.00 | -0.0050 | 0.600 | -66.95 | -0.0054 | | 0.249 | |
| *HCHS_20%* | | | | | | | | |
| Compositional effect | | | | | | | | |
| Total | 19.77 | 0.0028 | 0.001 | 25.37 | 0.0020 | | 0.004 | |
| Socio-demographic factors | 4.43 | 0.0006 |  | -4.18 | -0.0003 | |  | |
| Head: female | -1.60 | -0.0002 | 0.030 | 1.14 | 0.0001 | | 0.004 | |
| Head: no education | 4.88 | 0.0007 | 0.052 | 1.02 | 0.0001 | | 0.672 | |
| Rural | 1.15 | 0.0002 | 0.799 | -6.35 | -0.0005 | | 0.117 | |
| Health needs | 3.52 | 0.0005 |  | 14.65 | 0.0012 | |  | |
| Head: age group <30 | -0.17 | 0.0000 | 0.923 | -0.29 | 0.0000 | | 0.898 | |
| Head: age group 30–50 | 2.10 | 0.0003 | 0.060 | 2.09 | 0.0002 | | 0.255 | |
| Head: age group >50 | -0.34 | 0.0000 | 0.109 | 0.28 | 0.0000 | | 0.265 | |
| Household having children | 6.97 | 0.0010 | 0.000 | 18.24 | 0.0015 | | 0.000 | |
| Household having disabled people | 1.66 | 0.0002 | 0.001 | 2.18 | 0.0002 | | 0.069 | |
| Household size | -6.69 | -0.0009 | 0.014 | -7.85 | -0.0006 | | 0.221 | |
| Health insurance (household) | 10.81 | 0.0015 | 0.000 | 15.00 | 0.0012 | | 0.000 | |
| Travel time to health center (>0.5 hour) | 1.01 | 0.0001 | 0.534 | -0.10 | 0.0000 | | 0.970 | |
| Response effect | | | | | | | | |
| Total | 80.23 | 0.0112 | 0.000 | 74.63 | 0.0060 | | 0.000 | |
| Socio-demographic factors | 18.34 | 0.0026 |  | 16.15 | 0.0013 | |  | |
| Head: female | -3.31 | -0.0005 | 0.572 | 6.06 | 0.0005 | | 0.331 | |
| Head: no education | 1.73 | 0.0002 | 0.747 | 3.29 | 0.0003 | | 0.599 | |
| Rural | 19.93 | 0.0028 | 0.217 | 6.80 | 0.0005 | | 0.743 | |
| Health needs | 22.33 | 0.0031 |  | 64.96 | 0.0052 | |  | |
| Head: age group <30 | 3.71 | 0.0005 | 0.390 | 2.66 | 0.0002 | | 0.547 | |
| Head: age group 30–50 | 6.52 | 0.0009 | 0.199 | 4.63 | 0.0004 | | 0.387 | |
| Head: age group >50 | -8.60 | -0.0012 | 0.072 | -7.58 | -0.0006 | | 0.203 | |
| Household having children | -22.31 | -0.0031 | 0.046 | -8.82 | -0.0007 | | 0.421 | |
| Household having disabled people | 1.80 | 0.0003 | 0.572 | -7.20 | -0.0006 | | 0.050 | |
| Household size | 41.21 | 0.0058 | 0.059 | 81.27 | 0.0065 | | 0.001 | |
| Health insurance (household) | 28.48 | 0.0040 | 0.001 | 15.68 | 0.0013 | | 0.247 | |
| Travel time to health center (>0.5 hour) | 7.94 | 0.0011 | 0.533 | 6.07 | 0.0005 | | 0.647 | |
| Constant | 3.14 | 0.0004 | 0.920 | -28.23 | -0.0023 | | 0.447 | |
| *HCHS_30%* | | | | | | | | |
| Compositional effect | | | | | | | | |
| Total | 22.40 | 0.0040 | 0.000 | 21.02 | 0.0015 | | 0.013 | |
| Socio-demographic factors | 8.20 | 0.0015 |  | -2.91 | -0.0002 | |  | |
| Head: female | -1.36 | -0.0002 | 0.036 | 1.31 | 0.0001 | | 0.000 | |
| Head: no education | 4.78 | 0.0009 | 0.030 | 2.23 | 0.0002 | | 0.316 | |
| Rural | 4.78 | 0.0009 | 0.248 | -6.45 | -0.0005 | | 0.076 | |
| Health needs | 4.27 | 0.0008 |  | 13.69 | 0.0010 | |  | |
| Head: age group <30 | 1.89 | 0.0003 | 0.233 | -1.68 | -0.0001 | | 0.428 | |
| Head: age group 30–50 | 2.77 | 0.0005 | 0.005 | 1.40 | 0.0001 | | 0.416 | |
| Head: age group >50 | -0.17 | 0.0000 | 0.369 | 0.39 | 0.0000 | | 0.105 | |
| Household having children | 7.99 | 0.0014 | 0.000 | 17.42 | 0.0012 | | 0.000 | |
| Household having disabled people | 1.31 | 0.0002 | 0.004 | 2.02 | 0.0001 | | 0.068 | |
| Household size | -9.52 | -0.0017 | 0.000 | -5.87 | -0.0004 | | 0.321 | |
| Health insurance (household) | 9.04 | 0.0016 | 0.000 | 11.35 | 0.0008 | | 0.001 | |
| Travel time to health center (>0.5 hour) | 0.90 | 0.0002 | 0.535 | -1.12 | -0.0001 | | 0.656 | |
| Response effect | | | | | | | | |
| Total | 77.60 | 0.0140 | 0.000 | 78.98 | 0.0055 | | 0.000 | |
| Socio-demographic factors | 11.60 | 0.0021 |  | 22.71 | 0.0016 | |  | |
| Head: female | -1.49 | -0.0003 | 0.768 | 7.62 | 0.0005 | | 0.189 | |
| Head: no education | -2.79 | -0.0005 | 0.558 | 7.25 | 0.0005 | | 0.220 | |
| Rural | 15.88 | 0.0029 | 0.282 | 7.84 | 0.0005 | | 0.664 | |
| Health needs | 22.97 | 0.0041 |  | 71.08 | 0.0050 | |  | |
| Head: age group <30 | 0.05 | 0.0000 | 0.989 | 4.59 | 0.0003 | | 0.255 | |
| Head: age group 30–50 | 8.44 | 0.0015 | 0.073 | 5.35 | 0.0004 | | 0.284 | |
| Head: age group >50 | -6.25 | -0.0011 | 0.146 | -10.79 | -0.0008 | | 0.055 | |
| Household having children | -11.33 | -0.0020 | 0.259 | -10.37 | -0.0007 | | 0.284 | |
| Household having disabled people | 4.37 | 0.0008 | 0.165 | -5.46 | -0.0004 | | 0.118 | |
| Household size | 27.70 | 0.0050 | 0.158 | 87.76 | 0.0061 | | 0.000 | |
| Health insurance (household) | 24.12 | 0.0043 | 0.002 | 15.95 | 0.0011 | | 0.215 | |
| Travel time to health center (>0.5 hour) | 3.25 | 0.0006 | 0.776 | -0.64 | 0.0000 | | 0.958 | |
| Constant | 15.65 | 0.0028 | 0.568 | -30.12 | -0.0021 | | 0.357 | |
| *HCHS_40%* | | | | | | |  | |
| Compositional effect | | | | | | | | |
| Total | 21.18 | 0.0034 | 0.000 | 17.33 | | 0.0012 | | 0.032 |
| Socio-demographic factors | 7.92 | 0.0013 |  | -3.21 | | -0.0002 | |  |
| Head: female | -1.22 | -0.0002 | 0.043 | 1.15 | | 0.0001 | | 0.001 |
| Head: no education | 3.96 | 0.0006 | 0.054 | 1.75 | | 0.0001 | | 0.412 |
| Rural | 5.18 | 0.0008 | 0.190 | -6.11 | | -0.0004 | | 0.075 |
| Health needs | 3.70 | 0.0006 |  | 10.62 | | 0.0007 | |  |
| Head: age group <30 | 1.88 | 0.0003 | 0.204 | -1.46 | | -0.0001 | | 0.470 |
| Head: age group 30–50 | 1.97 | 0.0003 | 0.029 | 1.30 | | 0.0001 | | 0.430 |
| Head: age group >50 | 0.05 | 0.0000 | 0.766 | 0.35 | | 0.0000 | | 0.125 |
| Household having children | 7.54 | 0.0012 | 0.000 | 15.59 | | 0.0011 | | 0.000 |
| Household having disabled people | 1.14 | 0.0002 | 0.007 | 1.81 | | 0.0001 | | 0.088 |
| Household size | -8.87 | -0.0014 | 0.000 | -6.98 | | -0.0005 | | 0.232 |
| Health insurance (household) | 8.45 | 0.0014 | 0.000 | 10.36 | | 0.0007 | | 0.002 |
| Travel time to health center (>0.5 hour) | 1.21 | 0.0002 | 0.372 | -0.44 | | 0.0000 | | 0.854 |
| Response effect | | | | | | | | |
| Total | 78.82 | 0.0126 | 0.000 | 82.67 | | 0.0058 | | 0.000 |
| Socio-demographic factors | 21.32 | 0.0034 |  | 13.41 | | 0.0009 | |  |
| Head: female | -3.03 | -0.0005 | 0.531 | 6.62 | | 0.0005 | | 0.219 |
| Head: no education | -3.37 | -0.0005 | 0.487 | 5.61 | | 0.0004 | | 0.335 |
| Rural | 27.72 | 0.0044 | 0.044 | 1.19 | | 0.0001 | | 0.942 |
| Health needs | 24.51 | 0.0039 |  | 71.57 | | 0.0050 | |  |
| Head: age group <30 | -1.52 | -0.0002 | 0.661 | 5.54 | | 0.0004 | | 0.140 |
| Head: age group 30–50 | 6.70 | 0.0011 | 0.157 | 1.30 | | 0.0001 | | 0.778 |
| Head: age group >50 | -3.37 | -0.0005 | 0.413 | -8.55 | | -0.0006 | | 0.119 |
| Household having children | -12.89 | -0.0021 | 0.158 | -9.01 | | -0.0006 | | 0.305 |
| Household having disabled people | 3.41 | 0.0005 | 0.281 | -5.01 | | -0.0004 | | 0.137 |
| Household size | 32.17 | 0.0051 | 0.109 | 87.30 | | 0.0061 | | 0.000 |
| Health insurance (household) | 13.20 | 0.0021 | 0.095 | 15.28 | | 0.0011 | | 0.220 |
| Travel time to health center (>0.5 hour) | -1.49 | -0.0002 | 0.893 | 3.12 | | 0.0002 | | 0.780 |
| Constant | 21.29 | 0.0034 | 0.431 | -20.71 | | -0.0014 | | 0.498 |

**Figure S1. Absolute inequalities in HCHS using different thresholds in 2005 and 2010**

**Figure S2. Decomposing absolute inequality in HCHS using different thresholds by poverty status in 2005 and 2010**
